# Reduced Human Leukocyte Antigen (HLA) Protection in Gulf War Illness (GWI)

**DOI:** 10.1016/j.ebiom.2015.11.037

**Published:** 2015-11-22

**Authors:** Apostolos P. Georgopoulos, Lisa M. James, Margaret Y. Mahan, Jasmine Joseph, Angeliki Georgopoulos, Brian E. Engdahl

**Affiliations:** a Brain Sciences Center, Department of Veterans Affairs Health Care System, Minneapolis, MN 55417, USA; b Department of Neuroscience, University of Minnesota Medical School, Minneapolis, MN 55455, USA; c Department of Neurology, University of Minnesota Medical School, Minneapolis, MN 55455, USA; d Department of Psychiatry, University of Minnesota Medical School, Minneapolis, MN 55455, USA; e Center for Cognitive Sciences, University of Minnesota, Minneapolis, MN 55455, USA; f Graduate Program in Biomedical Informatics and Computational Biology, University of Minnesota, Minneapolis, MN 55455, USA; g Metabolic Service, Department of Medicine, Department of Veterans Affairs Health Care System, Minneapolis, MN 55417, USA; h Department of Medicine, University of Minnesota Medical School, Minneapolis, MN 55455, USA; i Department of Psychology, University of Minnesota, Minneapolis, MN 55455, USA

**Keywords:** Gulf War Illness (GWI), Human Leukocyte Antigen (HLA), Genetic risk, Veterans

## Abstract

**Background:**

Gulf War Illness (GWI) is a disease of unknown etiology with symptoms suggesting the involvement of an immune process. Here we tested the hypothesis that Human Leukocyte Antigen (HLA) composition might differ between veterans with and without GWI.

**Methods:**

We identified 144 unique alleles of Class I and II HLA genes in 82 veterans (66 with and 16 without GWI). We tested the hypothesis that a subset of HLA alleles may classify veterans in their respective group using a stepwise linear discriminant analysis. In addition, each participant rated symptom severity in 6 domains according to established GWI criteria, and an overall symptom severity was calculated.

**Findings:**

We found 6 Class II alleles that classified participants 84.1% correctly (13/16 control and 56/66 GWI). The number of copies of the 6 alleles was significantly higher in the control group, suggesting a protective role. This was supported by a significant negative dependence of overall symptom severity on the number of allele copies, such that symptom severity was lower in participants with larger numbers of allele copies.

**Interpretation:**

These results indicate a reduced HLA protection (i.e. genetic susceptibility) in veterans with GWI.

**Funding:**

University of Minnesota and U.S. Department of Veterans Affairs.

## Highlights

• Differences in Human Leukocyte Antigen distinguished veterans with Gulf War Illness (GWI) from healthy Gulf War era veterans.
• Veterans with GWI show genetic susceptibility which is associated with increased severity of hallmark symptoms of GWI.

A large number of Gulf War veterans suffer from diffuse and debilitating symptoms that resemble altered immune functioning. This study evaluated whether the Human Leukocyte Antigen (HLA) gene, which is central to immune functioning, differs between veterans with GWI and unaffected Gulf War veterans. The findings highlight robust differences in HLA composition between the two groups and suggest that veterans with GWI are characterized by genetic susceptibility that confers risk for typical GWI symptoms. These findings provide compelling genetic evidence of immune dysfunction in GWI.

## 1 Introduction

Shortly after the Gulf War (1990–91), veterans started to report a variety of health problems that began during, or soon after returning from, deployment, prompting investigation into the epidemiology and etiology of the complaints. Those investigations revealed that diffuse symptoms such as fatigue, musculoskeletal pain, mood and neurocognitive complaints, gastrointestinal problems, and rashes were most commonly reported. The constellation of symptoms, now commonly referred to as Gulf War Illness (GWI), has affected a substantial number of Gulf War veterans (Fukuda et al., 1998, Institute of Medicine National Research Council, 2006, Institute of Medicine National Research Council, 2010, Kang et al., 2009, Steele, 2000). Several population-based studies have demonstrated that these symptoms occur at significantly higher rates in deployed Gulf War veterans relative to their nondeployed peers and other veterans (Fukuda et al., 1998, Kelsall et al., 2009, Unwin et al., 1999), raising the issue about possible in-theater exposures and stress as contributing factors. However, these symptoms are also present in non-deployed military personnel (Steele, 2000), leading some to suspect other causes, including reactions to vaccine adjuvants (Israeli, 2012, Toubi, 2012). In summary, GWI is now a recognized constellation of symptoms of unclear etiology, also co-occurring with psychiatric disorders.

To date, the pathophysiology of GWI remains poorly understood. The overlap of GWI symptoms with symptoms of autoimmune disorders (e.g., chronic fatigue), together with evidence of abnormal immune activation following exercise challenge (Broderick et al., 2012, Broderick et al., 2013), raise the possibility that GWI reflects an abnormal immune process (Broderick et al., 2012, Broderick et al., 2013, Hotopf et al., 2000, Israeli, 2012, Moss, 2013, Toubi, 2012). This probable involvement of altered immune mechanisms prompted our investigation of Human Leukocyte Antigen (HLA) in GWI.

HLA genes are located in the Major Histocompatibility Complex (MHC) of chromosome 6 and play a central role in immune recognition (Meuer et al., 1982). Most investigations of association of HLA to various diseases have focused on evaluating HLA allele frequencies in diseases of interest, as compared to the general, healthy population. Such studies have demonstrated HLA involvement with cancer, autoimmune, and infectious diseases (Rioux et al., 2009, Trowsdale and Knight, 2013). HLA Class I proteins (HLA-A, B, C) are expressed on all nucleated cells and present peptides from endogenous proteins to cytotoxic T lymphocytes engaged in immune surveillance. HLA Class II proteins (HLA-DRB1, DRB3/4/5, DQB1, DPB1) are expressed on antigen-presenting cells and present peptides derived from exogenous proteins to CD4 + helper T cells (Reche and Reinherz, 2003). A previous study of Gulf War syndrome in 27 veterans found that HLA DRB1*15 was more prevalent in cases than controls with an odds ratio of 1.66, although this association was not statistically significant (O'Bryan et al., 2003).

Here we tested the hypothesis that HLA may be a contributing factor in developing GWI by performing a stepwise linear discriminant analysis which assessed how well the number of copies of HLA alleles could classify participants in their respective group.

## 2 Materials and Methods

### 2.1 Study Participants

Veterans Affairs (VA) medical records were reviewed to identify potential participants. Participants were recruited if they had completed a Gulf War physical or if they had served during the Persian Gulf War. Exclusionary criteria included central nervous system disorders (e.g. Parkinson's disease, dementia, cerebral vascular accidents, a history of traumatic brain injury, etc.), lifetime psychotic or bipolar diagnoses, and current drug dependence. Veterans who might have had difficulty understanding the protocol were also excluded from recruitment. Participants provided written informed consent prior to participation and were compensated for their time. The study protocol was approved by the Institutional Review Board at the Minneapolis VA Health Care System.

We studied a total of 82 participants, 16 controls (15 men, 1 woman; age range 43–71 years; 54.9 ± 10.2 years, mean ± SD) and 66 GWI (64 men, 2 women; age range 39–76 years; 50.6 ± 7.9 years). The mean age did not differ significantly between the 2 groups (P = 0.13, t-test). Participants completed a symptom presence/severity questionnaire developed for use in Kansas Gulf War veterans (Steele, 2000) that evaluates a range of symptoms associated with GWI and permits determination of case status according to either the Centers for Disease Control and Prevention (CDC) criteria (Fukuda et al., 1998) or the Kansas GWI case definition (Steele, 2000). Participants meeting either set of Gulf War case definition criteria were included in the study. All participants had deployed during the Gulf War and were free of any autoimmune disease.

The questionnaire asks participants to indicate if they have had a persistent problem over the last 6 months with various symptoms from the following six domains: fatigue, pain, neurological-cognitive-mood, skin, respiratory, and gastrointestinal. For each symptom rated as present, participants are asked to rate the severity of the symptom as mild, moderate, or severe, and to indicate whether the symptom first became problematic before, during or after deployment to the Gulf. Only symptoms that began during or after Gulf War service are counted toward diagnosis. There were six symptom domains: fatigue, pain, neurological-cognitive-mood, skin, gastrointestinal, and respiratory (Steele, 2000). Individual symptom severity was reported in a scale from 0 to 3. For each participant, an average score per domain was calculated, and a grand average across domains was used in the regression analysis below.

### 2.2 HLA Genotyping

DNA isolation was carried out from 3 ml of whole blood drawn in EDTA tubes, using a commercially available kit (ArchivePure cat. 2,300,730) from 5Prime (distributed by Fisher Scientific or VWR) with an expected yield of 50-150 μg of DNA. The purified DNA samples were sent to Histogenetics (http://www.histogenetics.com/) for high-resolution HLA Sequence-based Typing (SBT; details are given in https://bioinformatics.bethematchclinical.org/HLA-Resources/HLA-Typing/High-Resolution-Typing-Procedures/ and https://bioinformatics.bethematchclinical.org/WorkArea/DownloadAsset.aspx?id=6482). Their sequencing DNA templates are produced by locus- and group-specific amplifications that include exon 2 and 3 for class I (A, B, C) and exon 2 for class II (DRB1, DRB3/4/5, DQB1, and DPB1) and reported as Antigen Recognition Site (ARS) alleles as per ASHI recommendation (Cano et al., 2007).

### 2.3 Data Analysis

Standard statistical tests were used to analyze the data using the IBM-SPSS statistical package (version 23) and ad hoc Fortran computer programs employing the International Mathematics and Statistics Library (IMSL; Rogue Wave Software, Louisville, CO, USA) statistical and mathematical libraries. We performed a stepwise linear discriminant analysis to identify those alleles that would correctly classify participants to their respective groups (control, GWI) using the SPSS statistical package above. In that analysis, group membership was the classification variable and the numbers of copies for each allele for each participant were the predictor variables (N = 144 alleles). This analysis identified 6 alleles that, as a group, classified correctly 84.1% of the participants (see below). We validated the robustness of this allele set by calculating the classification rate using the leave-one-out method in the original sample and by performing an extensive analysis based on the bootstrap (Efron and Tibshirani, 1993), as follows. Each participant was classified 10,000 times using bootstrap samples of participants and associated data from the 6 alleles above as predictors. Specifically, each bootstrap sample was generated by random sampling with replacement from each one of the 2 groups (control and GWI) to a total of N = 200 participant values per group (in separate additional analysis, bootstrap samples of N = 100 participants were used). We then performed a linear discriminant classification analysis to classify the given participant to one of the 2 groups, based on the 2 bootstrapped participant samples. Finally, we calculated the correct classification rate for each group. The quality and strength of classification was assessed using standard methods, including chi-square analysis, calculating confidence intervals on the sensitivity and specificity using the binomial theorem and Wilson's conservative test (Wilson, 1927), and calculating the receiver operating characteristic (ROC) curve and its associated statistics.

A different issue concerns the possible effect of unequal group sizes on the results of the discriminant analysis (Sanchez, 1974). Ideally, the group sizes should be equal, or as closely equal as possible, because, then, the null hypothesis for chance classification is the same or very similar across groups. To remedy this sample inequality, Sanchez (1974) proposed the following procedure aimed to perform the discriminant analysis using the same (or very similar) sample sizes. Let *G*1 and *G*2 be two groups to be discriminated with sample sizes *m* and *km*, respectively. The standard discriminant analysis would be somewhat hampered by the unequal sample sizes, *km* > *m*. To remedy this, Sanchez (1974) proposed that, instead of a single discriminant analysis, *k* such analyses be performed, where one group is always the same *N*(*G*1) = *m*, whereas the other group is a random subsample (without replacement) from *G*2, of size $N\left(G2\right)=\frac{\mathrm{km}}{k}=m$, i.e. the same to that of *G*1, with equal chance probabilities of 0.5. The final classification rate is the average of the *k* classification outcomes. We applied this approach to our data as follows. The sample size of the control group was 16, and that of the GWI was 66. Since 66 cannot be divided exactly by 16, we performed the following steps.

*Step 1*: The original control group (N = 16) was used always as one of the two groups to be classified.
*Step 2*: The original GWI group (N = 66) was randomly permuted.
*Step 3*: Four consecutive subsamples of n = 16 each were identified and four discriminant analyses performed between the original control group and each subsample above. This yielded four classification rates the average of which provided an overall classification rate against a chance probability of 0.5, since sample sizes were equal.
*Step 4*: This procedure left out two participants from the GWI group. To remedy this, we performed Steps 2 and 3 10,000 times, each with a different permutation. In this way, all GWI participants contributed to the analysis.
*Step 5*: The final classification rate was derived as the average of the 10,000 rates above and its value assessed against the expected chance rate of 0.5 (50%).

We assessed the direction of the effect of those alleles (protective vs. predisposing) by carrying out four additional analyses. First, we compared the frequencies of those alleles between the two groups using a paired t-test. Second, we calculated the odds ratio ($\hat{\omega }$) for each allele, took its natural logarithm, ln($\hat{\omega }$), and tested the null hypothesis that its average does not differ significantly from zero, using a one-sample t-test. (If any cell in the 2 × 2 table had zero counts, the odds ratio was calculated after the constant 0.5 was added to all counts in the table, to avoid taking the logarithm of zero or dividing by zero.) Third, we performed a linear regression analysis where the overall symptom severity was the dependent variable and the number of copies of the 6 alleles was the independent variable; this analysis was also performed separately for each symptom domain. Significantly lower average allele frequencies in GWI, a significant negative ln($\hat{\omega }$) and a significant negative slope (and correlation) in the regression analysis would argue for a collective protective effect of the 6 alleles. Finally, we compared the frequencies of those 6 alleles in our two groups (GWI, controls) to the frequencies that have been reported in the literature. Specifically, we searched the website “Allele*Frequencies in Worldwide Populations” (http://www.allelefrequencies.net/) and identified the following three databases on Caucasian populations from the United States with 4-digit HLA genotyping of the 3 relevant Class II loci (DQB1, DPB1, DRB1): (1) “USA San Francisco Caucasian” (SF; N = 220; Skibola et al., 2012); (2) “USA Minnesota Olmsted” (MN; N = 339; Ovsyannikova et al., 2005); and (3) “USA Caucasian pop 5” (source: Center for Disease Control, CDC; N = 268; Rossman et al., 2003). Analyses and comparisons with our data were carried out separately for each one of the 3 databases above.

### 2.4 Role of the Funding Source

Partial funding for this study was provided by the US Department of Veterans Affairs (Service Directed Research Program, Project Number 3106) and the University of Minnesota (Brain and Genomics Fund [University of Minnesota Foundation Fund Number 20507] and the American Legion Brain Sciences Chair [University of Minnesota Foundation Fund Number 20442]). The sponsors had no role in the current study design, analysis or interpretation, or in the writing of this paper. The contents do not represent the views of the U.S. Department of Veterans Affairs or the United States Government.

## 3 Results

A total of 144 unique HLA alleles (74 Class I and 70 Class II, Table 1) were identified from a total of 82 participants. The stepwise linear discriminant analysis identified 6 alleles that yielded 84.1% correct classification of the 82 subjects to their respective groups. These results were robust. The leave-one-out classification rate for the original sample was 79.1% and for the 2 bootstrap analyses > 80% (80.1% for bootstrap samples of N = 100 and 81.2% for N = 200). We also performed the same stepwise discriminant analysis in a gender-homogeneous sample for men only (N = 79). The correct classification rate was 86.1% and the leave-one-out classification rate was 81.0%. The overall classification rate obtained by applying the procedure proposed by Sanchez (1974) (see Methods) was 82.3% (range: 78.1%–85.9%, N = 10,000 permutations), which is way substantially and consistently above the expected chance rate of 50%.

**Fig. 1:**
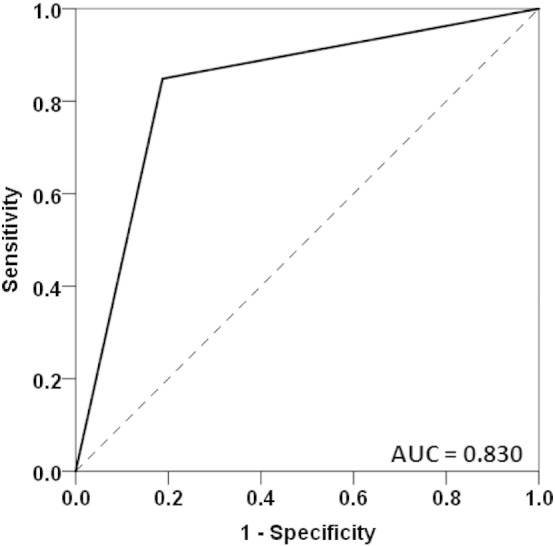
ROC curve of classification by the six discriminating alleles. AUC, Area Under the Curve. The asymptotic standard error of the AUC was 0.062; P = 0.00004; 95% confidence intervals for the AUC: 0.708–0.953.

The alleles and associated statistics are given in Table 2 and details about the goodness of classification based on the results using the full GWI sample are presented in Table 3. It can be seen that the discriminant classification was highly statistically significant and effective, and that the classification was correct at a high level (> 80%), was very similar with respect to sensitivity (0.848; 84.8%) and specificity (0.812; 81.2%), exceeded chance even by the most conservative Wilson's test (Table 3E), and yielded a ROC curve (Fig. 1) that was highly significant and considerably above chance (Table 3F).

The analysis of frequencies of the 6 alleles (Table 4, A, B) pointed to a systematic protective effect in the control group and, by extension, lack of protection in the GWI group. This is evidenced by the fact that all allele frequencies were lower in the GWI group (Table 4A), as compared to the control group (t = − 5.789, DF = 5, P = 0.002, paired t-test) and that all the odds ratios were less than one, i.e. negative ln($\hat{\omega }$): ln($\hat{\omega }$) = − 1.792 ± 0.383 (mean ± SEM), t = − 4.671, DF = 5, P = 0.005, one-sample t-test against the null hypothesis that mean ln($\hat{\omega }$) = 0). In addition, the percentage of participants with a given allele was systematically lower for all 6 alleles (Table 4B).

**Fig. 2:**
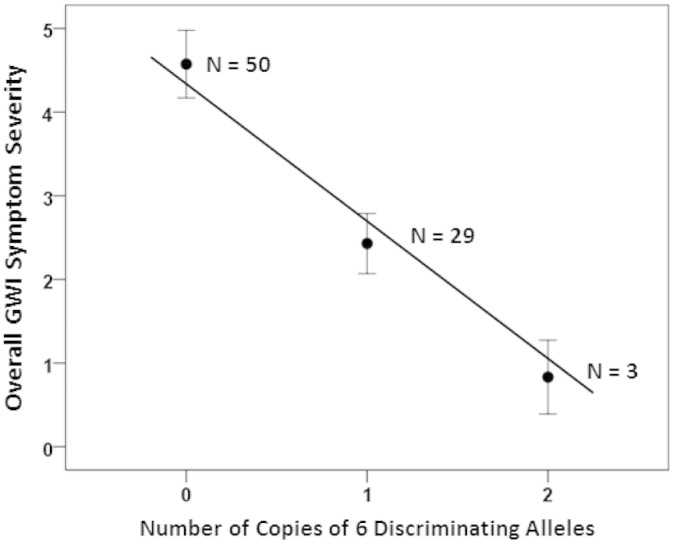
Mean GWI symptom severity is plotted against the average number of copies of the six discriminating alleles. N denotes the number of participants. (See text for details.)

This collective protective effect of the 6 alleles above was further corroborated by the results of the linear regression of overall symptom severity against the number of allele copies (Fig. 2) which revealed a strong and highly significant negative relation (t = − 4.148, DF = 80, P = 0.000083, R^2^ = 0.177):

(1) GWIsymptomseverity=4.55−2.044numberof6allelecopies

We also performed a multiple linear regression analysis separately for each symptom domain. We found a highly significant effect of the number of copies of individual alleles on symptom severity of Pain (P = 0.01, R^2^ = 0.199), Fatigue (P = 0.006, R^2^ = 0.210), and Neurological-Cognitive-Mood (P = 0.004, R^2^ = 0.225) symptoms; remarkably, all slopes (i.e. partial regression coefficients) of individual allele copies vs. symptom severity were negative in each regression model, indicating a consistent and robust effect. Finally, the regression analysis was not significant for skin (P = 0.911), gastrointestinal (P = 0.576), and respiratory (P = 0.598) symptoms.

**Fig. 3:**
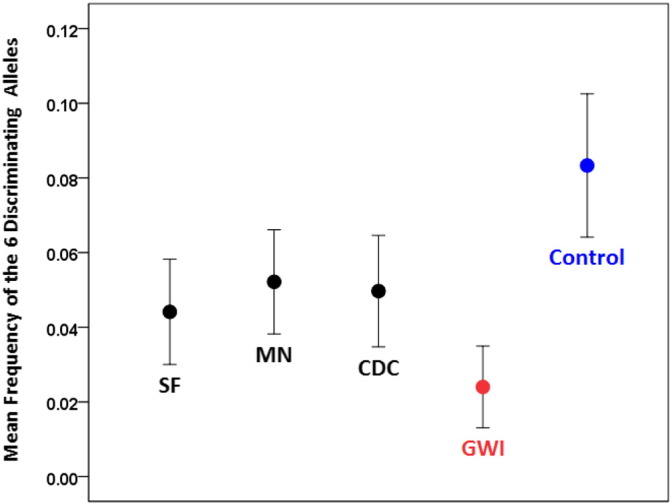
Mean (± SEM, N = 6 alleles) frequency of the six discriminating alleles (combined) for the five groups indicated.

**Fig. 4:**
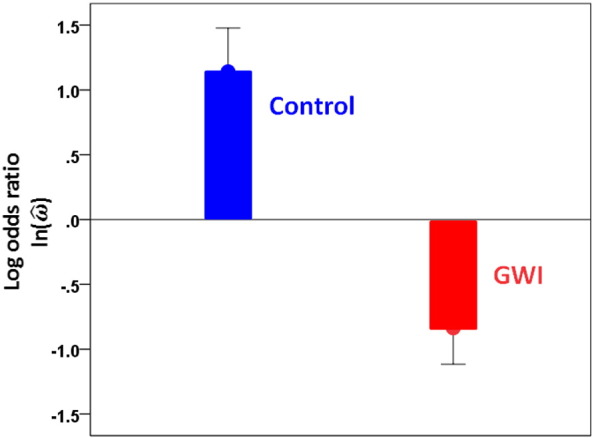
Mean ln($\hat{\omega }$) (± SEM) for the control and GWI groups across the three databases. (See text for details.)

**Fig. 5:**
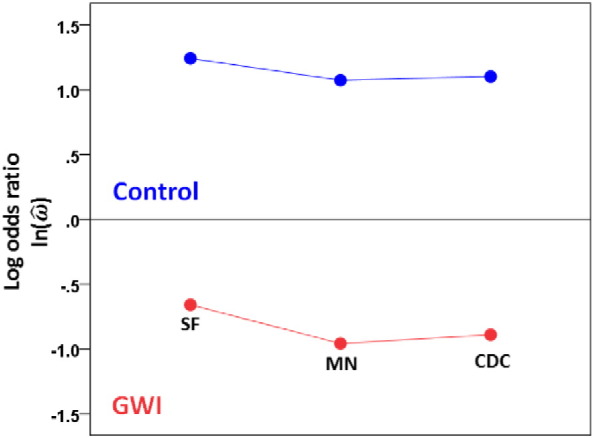
Mean ln($\hat{\omega }$) for the control and GWI groups for each database. (See text for details.)

Next, we analyzed our data with respect to the three databases (SF, MN, CDC) from the literature. Fig. 3 shows the mean frequencies across the 6 alleles as found in the 3 databases, our controls and the GWI group. It can be seen that the values for the 3 databases are similar, whereas the values for the GWI and control groups are lower and higher, respectively, than those of the databases. Next, we carried out a statistical analysis of these data by calculating the ln($\hat{\omega }$) for each allele between each database and control, and each database and GWI populations. This yielded 6 alleles × 3 databases × 2 groups = 36 values. We assessed the main effects of Database and Group, and the Database × Group interactions by performing a univariate ANOVA. We found that the Group main effect was highly significant (Fig. 4; P = 0.00019), whereas neither the Database main effect (P = 0.911; data not shown) nor the Database × Group interaction (Fig. 5; P = 0.934) were statistically significant (F-test in the ANOVA). These results document the significant difference between the control (increased) and GWI (decreased) mean allele frequencies, relative to the 3 databases, while positing the similarity among the 3 databases and the similarity of the group effect across these databases.

Finally, we searched the “Allele*Frequencies in Worldwide Populations” (http://www.allelefrequencies.net/) website for known haplotypes related to the 6 discriminating alleles, within the 3 databases that we used. Haplotype information was available only for the SF database, as shown in Table 5. It can be seen that no identified haplotype contains more than one of the 6 discriminating alleles, indicating their unique contribution.

## 4 Discussion

These results document for the first time significant differences in the HLA makeup between veterans with GWI and Gulf War era veterans without it. All the evidence from this study points to an enhanced vulnerability (or lack of protection) of the GWI veterans and, conversely, a reduced vulnerability (or additional protection) of the healthy veterans who served in the Gulf War but did not suffer from GWI. Collectively, our findings are in keeping with other evidence for an immune dysfunction in GWI (Broderick et al., 2012, 2013; Craddock et al., 2015, Hotopf et al., 2000, Israeli, 2012, Moss, 2013, Toubi, 2012, Whistler et al., 2009), in addition to other factors, including inflammatory components (Broderick et al., 2012, 2013; Johnson et al., 2013), mitochondria dysfunction (Koslik et al., 2014) and genetic variants regarding butyrylcholinesterase enzyme activity (Steele et al., 2015). In fact, our findings provide a genetic susceptibility framework within which environmental triggers (e.g. vaccines, exposure to chemicals, stress, etc.) can be discussed, investigated and evaluated.

All six HLA alleles that were singled out by the discriminant analysis to classify successfully (Fig. 1) control and GWI participants belonged in Class II and came from all three major genes (DQB1, DPB1, DRB1). Two alleles (DQB1*02:02 and DRB1*08:11) were absent from the GWI population, and the frequencies of the remaining four were all lower in the GWI group than in the control group (Table 4A). In addition, the percentage of participants with any of the six alleles was lower than in the controls (Table 4B). These results, together, document the lower frequency of occurrence of these alleles in the GWI group, as compared to the control group. The results of the regression analysis further documented the protective association of those alleles, given the significant negative slope in Fig. 2. In addition, this analysis further differentiated this effect among specific symptom domains, since it showed highly significant overall protective effects for Pain, Fatigue, Neurological-Cognitive-Mood domains. Remarkably, the effect of each allele on symptom severity in each one of these domains was protective, as evidenced by the universally negative partial regression coefficients in those three separate regression analyses. In contrast, the effect was not significant for the Skin, Gastrointestinal and Respiratory domains.

The comparison of the observed allele frequencies to those reported in published databases (Ovsyannikova et al., 2005, Rossman et al., 2003, Skibola et al., 2012) (Fig. 3, Fig. 4, Fig. 5) documented an additional finding, namely that both GWI and controls differed systematically and in opposite directions (higher for controls, lower for GWI, Fig. 4, Fig. 5) with respect to the published allele frequencies. This suggests the following hypothesis regarding the role of the six discriminating alleles we identified. First, we assume that the population of GW-era veterans came from a larger population whose allele frequencies would be very similar to those reported in the three databases we used in this study. This is a reasonable assumption. Second, it is known that GW veterans were administered various vaccines, possibly together and/or multiply (Institute of Medicine National Research Council, 2000, Petersdorf et al., 1996, Schwartz et al., 1997, Steele, 2000) and they were exposed to various chemical agents (Institute of Medicine National Research Council, 2000, Steele et al., 2015). Third, a proportion of GW veterans developed GWI consisting of chronic symptoms affecting multiple organ systems (Fukuda et al., 1998, Institute of Medicine National Research Council, 2006, Institute of Medicine National Research Council, 2010, Kang et al., 2009, Kelsall et al., 2009, Steele, 2000), mostly affecting veterans who were deployed but also present in non-deployed or minimally exposed veterans (Steele, 2000). Based on those facts, we hypothesize that vaccinations and/or chemical exposures of GW era veterans served as environmental triggers (“hits”) that contributed to developing GWI in genetically (HLA) “vulnerable” veterans. Specifically, we propose that the presence of certain HLA alleles in higher frequencies conferred protection, whereas their relative paucity conferred vulnerability. This, in turn, resulted in the two distinct subpopulations of our study, namely control, deployed GW veterans with higher allele frequencies and absence of GWI on the one hand, and deployed GW veterans with lower frequencies and presence of GWI (Fig. 3). It should be noted that both vaccinations (Hotopf et al., 2000, Israeli, 2012, Toubi, 2012, Rook and Zumla, 1997) and chemical exposures (Moss, 2013) have been implicated previously as contributing factors for the development of GWI. The results of our study simply add a genetic susceptibility framework within which the effects of the factors above could be interpreted and investigated in future work. The nature of this postulated protection is likely to relate to autoimmune as well as inflammatory processes, since HLA has been implicated in both (Trowsdale and Knight, 2013, Johnson et al., 2013, Blackwell et al., 2009). In addition, HLA genetic underpinnings in immune responses to vaccines have been well established (Ovsyannikova et al., 2006, Ovsyannikova and Poland, 2011, Poland et al., 2007, Poland et al., 2008a, Poland et al., 2008b). Specific HLA makeup could thus manifest as autoimmune reactions, aberrant immune response to vaccinations, and/or increased susceptibility to infections (Nicolson, 1998); all three of them have been implicated in GWI.

A major limitation of our study is the small number of participants studied which, nevertheless, yielded systematic, significant and robust differences between the control and GWI groups but also between those two groups and published HLA allele databases. In this context, it is especially noteworthy that high correct classification rates were obtained in our extensive bootstrap analyses and permutation analyses (Sanchez, 1974) which documented the robustness of our results, i.e. their extension to larger sample populations. However, extensive bootstraps cannot substitute for validation in new, independent samples, which remains to be done. Finally, it is reasonable to assume that the six alleles identified in the present study are only part of a protective HLA makeup. We anticipate that a similar but large scale study will identify more such alleles and will provide a firm background to investigate the molecular mechanisms by which such protection/vulnerability may be mediated (Wahren-Herlenius and Dorner, 2013).

## Contributions

LMJ and BEE contributed to data collection and clinical evaluation; APG and JJ contributed to data analysis; MYM and AG contributed to DNA extraction and arranging for HLA genotyping; APG, LMJ, AG wrote the paper. All authors contributed to extensive editing of the paper.

## Declaration of interests

The authors do not report any financial disclosures or conflicts of interest.

## Figures and Tables

**Table 1:** Distribution of 144 HLA 4-digit resolution unique alleles identified to corresponding genes.

Class	Gene	Count	Percent
II	A	19	13.2
II	B	36	25.0
II	C	19	13.2
II	DRB1	29	20.1
II	DRB3	3	2.1
II	DRB4	1	0.7
II	DRB5	3	2.1
II	DQB1	15	10.4
II	DPB1	19	13.2
	Total	144	100.0

**Table 2:** The six HLA alleles identified by the stepwise linear discriminant analysis and associated statistics. All alleles are from Class II.

A. Stepwise statistics				
	Allele	Tolerance	F-to-remove	Wilks' Lambda
1	DQB1*02:02	0.934	12.942	0.749
2	DPB1*06:01	0.913	10.162	0.725
3	DRB1*13:02	0.872	11.446	0.736
4	DRB1*08:11	0.943	8.249	0.709
5	DRB1*01:01	0.904	5.329	0.684
6	DPB1*01:01	0.923	4.228	0.675


B. Canonical discriminant function statistics		
Eigenvalue	% of variance	Cumulative %
0.565	100	100


Wilks' Lambda	Chi-square	DF	Significance
0.639	34.5	6	0.000005

**Table 3:** Classification results.

A. Classification table				
		Predicted		Total
GWI	Control	
Observed	GWI	56	10	66
Control	3	13	16
Total		59	23	82


B. Analysis of the two-way classification table			
Test	Value	DF	Significance (2-sided)
Pearson Chi-Square (corrected for continuity)	27.9	1	P = 1.29 × 10^− 7^
Fisher's Exact Test			P = 9.25 × 10^− 7^


C. Mantel–Haenszel common odds ratio estimate			
Estimated odds ratio ($\hat{\omega }$)	ln($\hat{\omega }$)	SE of ln($\hat{\omega }$)	Asymptotic significance (2-sided)
24.267 95% lower bound: 5.840 95% upper bound: 100.83	3.189	0.727	P = 0.000011



D. Sensitivity, specificity, and binomial confidence intervals			
	Observed	95% confidence intervals	
	Lower bound	Upper bound
Sensitivity	0.848 (84.8%)	0.804	0.893
Specificity	0.812 (81.2%)	0.715	0.910


E. Sensitivity, specificity, and Wilson's (1927) confidence intervals			
	Observed	95% confidence intervals	
	Lower bound	Upper bound
Sensitivity	0.848	0.702	0.888
Specificity	0.812	0.570	0.934

**Table 4:** Frequency analysis of the 6 discriminating alleles.

A. Frequencies and odds ratios					
	Allele	GWI frequency	Control frequency	Estimated odds ratio ($\hat{\omega }$)	ln($\hat{\omega }$)
1	DQB1*02:02	0	0.0625	0.046^a^	− 3.078
2	DPB1*06:01	0.007576	0.0625	0.114	− 2.167
3	DRB1*13:02	0.0379	0.125	0.276	− 1.289
4	DRB1*08:11	0	0.0312	0.0792^a^	− 2.535
5	DRB1*01:01	0.0682	0.1562	0.395	− 0.927
6	DPB1*01:01	0.0303	0.0625	0.469	− 0.758


B. Percent of participants per group and allele			
	Allele	GWI	Control
1	DQB1*02:02	0	12.5
2	DPB1*06:01	1.5	12.5
3	DRB1*13:02	7.6	25
4	DRB1*08:11	0	6.3
5	DRB1*01:01	13.6	18.8
6	DPB1*01:01	6.1	12.5

a The odds ratio was estimated after adding 0.5 to all counts to avoid taking the logarithm of zero. This procedure underestimates the true effect.

**Table 5:** Haplotypes reported for the SF database at the “Allele*Frequencies in Worldwide Populations” website (http://www.allelefrequencies.net/). No haplotypes reported for alleles DPB1*06:01 and DRB1*08:11.

Haplotypes		Frequency (%)
A. DQB1*02:02		
1	DRB1*07:01-DQA1*02:01-DQB1*02:02	8.9
2	DRB1*07:01-DRB4*01:01-DQA1*02:01-DQB1*02:02-DPB1*04:01	2.4
3	DRB1*07:01-DRB4*01:01-DQA1*02:01-DQB1*02:02-DPB1*04:02	1.3
4	DRB1*07:01-DRB4*01:01-DQA1*02:01-DQB1*02:02-DPB1*11:01	1.1
5	DRB1*07:01-DRB4*01:01-DQA1*02:01-DQB1*02:02-DPB1*17:01	1.8

B. DRB1*13:02		
1	DRB1*13:02-DQA1*01:02-DQB1*06:04	3.0
2	DRB1*13:02-DQA1*01:02-DQB1*06:09	1.4
3	DRB1*13:02-DRB3*03:01-DQA1*01:02-DQB1*06:04-DPB1*04:01	1.3

C. DRB1*01:01		
1	DRB1*01:01-DQA1*01:01-DQB1*05:01	6.4

D. DPB1*01:01		
1	DRB1*03:01-DRB3*01:01-DQA1*05:01-DQB1*02:01-DPB1*01:01	3.90

## References

[bb0005] Blackwell J.M., Jamieson S.E., Burgner D. (2009). HLA and infectious diseases. Clin. Microbiol. Rev..

[bb0010] Broderick G., Ben-Hamo R., Vashishtha S. (2013). Altered immune pathway activity under exercise challenge in Gulf War Illness: an exploratory analysis. Brain Behav. Immun..

[bb0015] Broderick G., Fletcher M.A., Gallagher (2012). Exploring the diagnostic potential of immune biomarker coexpression in Gulf War Illness. Methods Mol. Biol..

[bb0020] Cano P., Klitz W., Mack S.J. (2007). Common and well-documented HLA alleles: report of the Ad-Hoc committee of the American society for histocompatiblity and immunogenetics. Hum. Immunol..

[bb0025] Craddock T.J., Harvey J.M., Nathanson L., Barnes Z.M., Klimas N.G., Fletcher M.A. (2015). Using gene expression signatures to identify novel treatment strategies in gulf war illness. BMC Med. Genomics.

[bb0030] Efron B., Tibshirani R. (1993). An Introduction to the Bootstrap.

[bb0035] Fukuda K., Nisenbaum R., Stewart G. (1998). Chronic multisymptom illness affecting Air Force veterans of the Gulf War. JAMA.

[bb0040] Hotopf M., David A., Hull L. (2000). Role of vaccinations as risk factors for ill health in veterans of the Gulf war: cross-sectional study. BMJ.

[bb0045] Institute of Medicine National Research Council (2000). Gulf War and Health: Volume 1. Depleted Uranium, Pyridostigmine Bromide, Sarin, and Vaccines.

[bb0050] Institute of Medicine National Research Council (2006). Gulf War and Health: Volume 4. Health Effects of Serving in the Gulf War.

[bb0055] Institute of Medicine National Research Council (2010). Gulf War and Health: Volume 8: Update of Health Effects of Serving in the Gulf War.

[bb0060] Israeli E. (2012). Gulf War Syndrome as a part of the autoimmune (autoinflammatory) syndrome induced by adjuvant (ASIA). Lupus.

[bb0065] Johnson G.J., Leis L.A., Slater B.C., Bach R.R. (2013). Elevated platelet count, C-reactive protein and thromboxane analog-induced platelet aggregation in patients with Gulf War veterans' illnesses: evidence of a chronic inflammatory state?. Blood Coagul. Fibrinolysis.

[bb0070] Kang H.K., Li B., Mahan C.M. (2009). Health of US veterans of 1991 Gulf War: a follow-up survey in 10 years. J. Occup. Environ. Med..

[bb0075] Kelsall H.L., McKenzie D.P., Sim M.R. (2009). Physical, psychological, and functional comorbidities of multisymptom illness in Australian male veterans of the 1991 Gulf War. Am. J. Epidemiol..

[bb0080] Koslik H.J., Hamilton G., Golomb B.A. (2014). Mitochondrial dysfunction in Gulf War illness revealed by ^31^Phosphorus Magnetic Resonance Spectroscopy: a case–control study. PLoS One.

[bb0085] Meuer S.C., Hussey R.E., Hodgdon J.C. (1982). Surface structures involved in target recognition by human cytotoxic T lymphocytes. Science.

[bb0090] Moss J.I. (2013). Gulf War illnesses are autoimmune illnesses caused by increased activity of the p38/MAPK pathway in CD4 + immune system cells, which was caused by nerve agent prophylaxis and adrenergic load. Med. Hypotheses.

[bb9100] Nicolson G.L. (1998). Chronic infections as a common etiology for many patients with Chronic Fatigue Syndrome, Fibromyalgia Syndrome and Gulf War Illnesses. Intern. J. Med..

[bb0095] O'Bryan T.A., Romano P.J., Zangwill B.C. (2003). Human leukocyte antigens in Gulf War veterans with chronic unexplained multiple symptoms. Mil. Med..

[bb0100] Ovsyannikova I.G., Poland G.A. (2011). Vaccinomics: current findings, challenges and novel approaches for vaccine development. AAPS J..

[bb9200] Ovsyannikova I.G., Jacobson R.M., Ryan J.E., Vierkant R.A., Pankratz V.S., Jacobsen S.J. (2005). HLA class II alleles and measles virus-specific cytokine immune response following two doses of measles vaccine. Immunogenetics.

[bb0105] Ovsyannikova I.G., Dhiman N., Jacobson R.M., Poland G.A. (2006). Human leukocyte antigen polymorphisms: variable humoral immune responses to viral vaccines. Expert Rev. Vaccines.

[bb0110] Petersdorf R.G., Page W.F., Thaul S. (1996). Committee to Study the Interactions of Drugs, Biologics, and Chemicals in US Military Forces Interactions of Drugs, Biologics, and Chemicals in US Military Forces.

[bb0115] Poland G.A., Ovsyannikova I.G., Jacobson R.M. (2008). Vaccine immunogenetics: bedside to bench to population. Vaccine.

[bb0120] Poland G.A., Ovsyannikova I.G., Jacobson R.M. (2008). Personalized vaccines: the emerging field of vaccinomics. Expert. Opin. Biol. Ther..

[bb0125] Poland G.A., Ovsyannikova I.G., Jacobson R.M., Smith D.I. (2007). Heterogeneity in vaccine immune response: the role of immunogenetics and the emerging field of vaccinomics. Clin. Pharmacol. Ther..

[bb0130] Reche P.A., Reinherz E.L. (2003). Sequence variability analysis of human class I and class II MHC molecules: functional and structural correlates of amino acid polymorphisms. J. Mol. Biol..

[bb0135] Rioux J.D., Goyette P. (2009). International MHC and Autoimmunity Genetics Network. Mapping of multiple susceptibility variants within the MHC region for 7 immune-mediated diseases. Proc. Natl. Acad. Sci. U. S. A..

[bb0140] Rook G.A., Zumla A. (1997). Gulf War syndrome: is it due to a systemic shift in cytokine balance towards a Th2 profile?. Lancet.

[bb0145] Rossman M.D., Thompson B., Frederick M., Maliarik M., Iannuzzi M.C., Rybicki B.A. (2003). HLA-DRB1*1101: a significant risk factor for sarcoidosis in blacks and whites. Am. J. Hum. Genet..

[bb0150] Sanchez P.M. (1974). The unequal group size problem in discriminant analysis. J. Acad. Mark. Sci..

[bb0155] Schwartz D.A., Doebbeling B.N., Merchant J.A., Barret D.H. (1997). Self-reported illness and health status among Gulf War veterans—a population-based study. J. Am. Med. Assoc..

[bb0160] Skibola C.F., Akers N.K., Conde L., Ladner M., Hawbecker S.K., Cohen F. (2012). Multi-locus HLA class I and II allele and haplotype associations with follicular lymphoma. Tissue Antigens.

[bb0165] Steele L. (2000). Prevalence and patterns of Gulf War illness in Kansas veterans: association of symptoms with characteristics of person, place, and time of military service. Am. J. Epidemiol..

[bb0170] Steele L., Lockridge O., Gerkovich M.M. (2015). Butyrylcholinesterase genotype and enzyme activity in relation to Gulf War illness: preliminary evidence of gene-exposure interaction from a case–control study of 1991 Gulf War veterans. Environ. Heal..

[bb0175] Toubi E. (2012). ASIA — Autoimmune syndromes induced by adjuvants: rare, but worth considering. IMAJ.

[bb0180] Trowsdale J., Knight J.C. (2013). Major histocompatibility complex genomics and human disease. Annu. Rev. Genomics Hum. Genet..

[bb0185] Unwin C., Blatchley N., Coker W. (1999). Health of UK servicemen who served in Persian Gulf War. Lancet.

[bb0190] Wahren-Herlenius M., Dorner T. (2013). Immunopathogenic mechanisms of systemic autoimmune disease. Lancet.

[bb0195] Whistler T., Fletcher M.A., Lonergan W. (2009). Impaired immune function in Gulf War Illness. BMC Med. Genomics.

[bb0200] Wilson E.B. (1927). Probable inference, the law of succession, and statistical inference. J. Am. Stat. Assoc..

